# Pathology

**Published:** 1825-04-01

**Authors:** 


### PATHOLOGY.

Actio læsa suum designat singula morbum;
Præterea morbum partis mutatio signat.

1. *Mortal Dyspnœa.** As pathology has made progress, the number of dyspnœas, said to be nervous, spasmodic, or unattended by any appreciable lesion of structure, have greatly diminished. But, although the greater number of asthmas can be traced to organic disease of either the vascular or respiratory systems, there are still cases where the cause of dyspnœa eludes the eye and scalpel of the anatomist.

*Case* 1. A baker, 20 years of age, of strong constitution, and resident in Paris only two months, became affected, five or six weeks before the date of this report, with a slight diarrhœa, which continued till the 10th of April, when he was seized with the precursory symptoms of measles. On the 14th, the eruption came out—and, on the 15th, it had covered the whole body. He was admitted into LA CHARITE in the evening of that day. The pulse was hard—the tongue and lips red—the cough severe, and, in short, the symptoms very alarming. Towards the middle of the night he experienced a sudden oppression about the chest, which augmented rapidly, and, on the morning of the 16th, he was found by the physicians in a state of demi-asphyxia—the eyes prominent, the face purple, the breathing short and difficult, cough incessant, scanty mucous expectoration. The chest sounded well, on percussion, throughout every part, and the stethoscope exhibited the "mucous rattle" in various points. The eruption had vanished, with the exception of a few pale spots. The pulse was quick and hard, the tongue red. This train of symptoms seemed to indicate pneumonia, but the *pathognomonic signs* of this inflammation were entirely wanting. "Could simple bronchitis of an extremely acute character and sudden rise, occasion such a severe state of dyspnœa? Could this phlegmasia, joined to phlegmasia of the mucous membrane of the alimentary canal, explain the state of extreme danger into which this patient had so suddenly fallen?" In whatever way these questions may be answered, says M. Andral, the indications to be pursued were not doubtful. The internal inflammation was to be reduced—and the eruption on the skin

* Andral Jun. Recueillie à l'Hopital de la Charité.—Revue Med. Sept. 1824.

was to be restored, if possible. Twenty leeches to each side of the chest, and ten to the epigastrium—a blister to the inside of each leg—skin rubbed with an ammoniacal embrocation. A great degree of relief followed these measures. In the evening, the breathing was much less oppressed—the cough less frequent—the tongue not so red—but the eruption was not restored. 17th. The symptoms of intense bronchitis, with proportionate fever, were still in existence. The breathing was but little accelerated. 18th. The fever almost subsided—and the opake appearance of the expectoration announced a favorable termination of the bronchitis. All at once, this evening, the breathing became greatly oppressed—and twelve ounces of blood were taken from the arm. 19th. The dyspnœa was still great—the pulse quicker. During the whole of this day the suffocation augmented more and more. 20th. Livor of the face and lips—shrinking of all external parts—death.

*Dissection.* The mucous membrane of the larynx, trachea, and bronchia, to their minutest ramifications, was of an intensely red colour. In some portions of the first divisions of the bronchia, there were appearances of incipient false membranes, similar to those of croup. The parenchymatous structure of the lungs was sound and crepitous throughout. Heart sound. The mucous membrane of the stomach and bowels was natural, with the exception of a red patch near the valve of the colon. The liver was gorged with blood—the spleen large and firm. There was considerable serous effusion under the tunica arachnoidea—but the substance of the brain was not injected—considerable effusion of serum into both ventricles.

We cannot participate in M. Andral's astonishment at the fatal termination of this case without organic disease of the parenchymatous structure of the lungs. Such an intense bronchial inflammation, as here presented itself, was quite sufficient to cause death. It is also to be remembered that those internal inflammations, succeeding repelled measles, are always more dangerous than inflammations of the same organs under common circumstances. We do not exactly understand why M. Andral should place the case in question under the head of mortal dyspnœa without any appreciable lesion—" sans lesion appreciable." Surely the state of the mucous membrane above described is an " appreciable lesion," in every sense of the word. We should certainly consider a structure which is in a high state of inflammation as one that is a state of *lesion* or alteration from the natural state. Let us hear how M. Nysten defines the word in his Medical Dictionary. " Lésion (from Lædo) se dit de *toute alteration* des proprietés vitales, des fonctions de l'economie animale, ou des tissues organiques." Here then we have every *alteration*, whether in function or structure, properly designated as a *lesion*.

*Case* 2. A man, 40 years of age, had had, for many years, a large ulcer on the left leg, which was greatly swelled beneath the ulcered part, the limb resembling a case of elephantiasis. From this ulcer much pus was daily discharged. For six or seven months past this man had also a moist cough, without dyspnœa or pain in any part of the thorax. While in hospital for the ulcered leg, he was suddenly seized with severe dyspnœa, at the same time it was remarked that the ulcer was nearly dry. The patient was seated on his bed in a state of inexpressible anxiety, conjuring the physician to relieve him of the load that weighed upon his chest, and threatened every moment to suffocate him. The pulse, which was moderately quick, was easily compressible—the inspirations were short, quick, as if by convulsive efforts. In vain did they seek for a cause of this alarming state, in the heart or lungs;—the structure of which, on minute examination, appeared sound. The cause of the dyspnœa, then, could not be ascertained—and the symptom became more formidable every hour. It was not alleviated by sanguineous depletion, blisters, or other remedies employed. Next morning the patient appeared to be in momentary peril of suffocation. With the hope that the cause might be situated in the larynx, tracheotomy was performed by M. Roux; but no alleviation followed, and the patient expired in the evening.

*Dissection.* The parenchymatous structure of the lungs was perfectly sound and crepitous, with the exception of a small hepatized portion in the left side. The mucous membrane exhibited only a few patches of redness, and those of trifling extent. The heart and great vessels were sound. Nothing remarkable in the head or abdomen.

In this case, we are forced to admit that the appearances, *post mortem*, were by no means such as could explain the fatal dyspnœa—and therefore we agree with M. Andral that—if ever there was a case of purely nervous or spasmodic asthma, unconnected with organic lesion, this was a fair specimen. It teaches us one important lesson (which seems to be little attended to in these days of ultra-pathology) that the *functions* of vital organs may be so far disturbed as to cause death, *before* any organic change takes place that remains cognizable after life has ceased. How many thousands of the profession are daily led astray by mistaking the *consequence* of disordered function for the *cause* of it? Had the above patient lived a few days longer, and until the effusion or other morbid effect took place, the cause of death would have been set down, unquestionably, to its account. It is but rarely, however, that we have opportunities of seeing life cut off by disordered function alone. Organic changes so generally obtain before the fatal event, that we are naturally led to connect them as cause and effect. It is in this way that pathology is capable of leading us into error. It is worthy of being remembered that the dyspnœa, in the above case, commenced about the time that the discharge from the ulcer ceased. There is but too much scepticism, in the present day, respecting the serious effects of suppressed discharges, repelled eruptions, and metastases of disordered action. We are sorry for this—and we think it may be accounted for by the attention of men being more directed to organic changes than to minute symptomatology—or, in other words, disturbed functions.

M. Andral mentions the outlines of a case which occurred in the practice of Dr. Bouillaud in the Hopital Cochin, and which bears on this subject. A man had been a long time affected with a dartrous eruption which suddenly disappeared without any known cause or particular application. At this precise juncture, the man became affected with dyspnœa, which soon became extreme, and with which he never had been affected previously. On minute examination, no organ could be found in fault. Leeches and blisters were applied to the part whence the eruption had disappeared. The dyspnœa, by this measure, was completely removed in a few hours.

M. Guersent, an accurate observer of phenomena, has related two cases of children, severely affected with remittent dyspnœa that proved fatal in both cases, and in a very prompt manner. The most minute anatomical observation could not detect a single organic lesion of any part in the body.

---

2. *Inflammation of Veins.** Inflammation is by no means a rare occurrence after bleeding, nor is *suppuration* itself so rare a disease as Dr. Chapman supposes. Our author can find but one case on record, analogous to his own, and that case is only sketched by John Hunter. In dissecting the arm of a man, Mr. Hunter traced a series of adhesions along the course of the vein. But near the axilla, says he, "the vein had taken on suppuration, beyond which adhesions had not formed, and this had given a free passage for the matter into the circulation, of which the patient most probably died." The case related by Dr. Chapman occurred in the person of a coachman, who, after being bled for a slight pleuritic complaint, continued to exercise the arm for several days after inflammation had commenced. When Dr. Chapman first saw him, the case presented a serious aspect. The arm was swelled to twice its natural size, the pain and inflammation being excessive. On pressure, a copious stream of purulent matter issued from the orifice, and the vein could be distinctly traced for several inches, in an enlarged state, imparting the sensation of a hard inelastic tube under the integuments. There was pain in the left side of the chest, and a universal soreness over the body. Little or no pyrexia was observable, but the pulse was quick, weak, and irregular. The patient was greatly harrassed by restlessness and agitation. There was also some incoherence of ideas. Dr. Chapman had no doubts as to the nature of the disease—" inflammation of the vein, extending probably to the heart, and the introduction of pus into the blood." "To subdue the inflammation of the vein, and arrest the pus in its passage to the circulation, were obvious indications." Notwithstanding the feebleness of the arterial action, our author bled to 12 ounces. The bleeding was followed by no sensible effect, and exhibited no phenomena of inflammation. The

---

* Dr. Chapman. Philadelphia Journal, No. 14, February, 1824. Art. Phlebite, by M. M. Breschet et Vilermé.—*Dict. des Sciences Medicales.*

serum and crassamentum continued commixed, as if slightly stirred together.

Here we would just put it to Dr. Chapman, whether *suppuration*, when once established in a part, can be *safely* checked by bleeding? Do we resort to this measure under common circumstances? Is not the character of an inflammation completely changed, *after* the suppurative process has once taken place? Dr. Chapman may say that he apprehended inflammation of the heart. The symptoms certainly did not resemble those of carditis. They much more resembled what we would be led to expect from a sedative substance taken into the circulation, and depressing all the powers of life.

But to the sequel of the case. The arm was enveloped in a blister, except at the puncture, to which a poultice was applied. Purgatives, till the bowels were freely opened. In the evening he seemed somewhat better; but, in the night, the pain in the left side returned with violence. "*It did not, however, raise his pulse.*" Our author became convinced that the pain arose from inflammation of the heart, and therefore placed a large blister over the region of that organ. Though relieved in respect to pain, our author, on the following morning, (second) had the mortification of finding the other symptoms worse. "With a pulse weak and tremulous, he was wild and distracted." Dr. Chapman again directed blood-letting by cupping to the amount of six or eight ounces, and a blister to be applied behind each ear. A bandage and compress were also applied, a short distance above the puncture, with the view of preventing the passage of pus along the vein. *Third morning*, still worse. The morbid sensibility of the whole body had become so great, that the patient could scarcely bear the weight of the bed-clothes. At this period Dr. James added his assistance, and they agreed to give camphorated emulsion in large doses. But all was unavailing—the patient sunk progressively, though by opium and strong stimulants life was preserved for two or three days longer.

*Dissection.* The vein was exposed from the wrist to the axilla—and this appears to be all that was permitted. The external surface of the vessel was, in many places, inflamed, and especially above the puncture. Betwixt this and the shoulder, matter had penetrated through the vein, by four distinct apertures, into the neighbouring cellular membrane, forming small abscesses. Directly around the puncture there was an abscess, containing a large spoonful of pus, mixed with a dark fetid sanies. Sphacelus had already destroyed a portion of the cellular texture. The vein laid open, inflammation, more or less, was seen from a little below the elbow to the final point of dissection. The appearance in the *coat* indicated the highest grade of inflammation; the appearances on the internal surface of the vessel were not so strong. Below and above the orifice, for several inches, gangrene had taken place, bounded by an extensive erysipelatous blush, and the inner surface of the vessel, within this space, had begun to slough. The quantity of pus in the cavity of the vein was small. The discharge of matter from the wound indeed had greatly diminished prior to the death of the patient. No appearances of adhesions presented themselves in any part of the vessel.

It is a great pity that Dr. Chapman was unable to prosecute the dissection farther, as the question of carditis was thereby left undecided. We think that, from the symptoms during the life of the patient, and from the general history of such cases (some of which we shall shortly notice,) the probability is, that inflammation had not reached the heart. In cases so untoward, and, comparatively speaking, so uncommon as these, the rule of conduct, in respect to treatment, must be left to the judgment of the practitioner. We can have no doubt that Dr. Chapman saw indications for the depletive measures which he put in force, nor have we the slightest idea of making any reflection on the treatment pursued. But we would venture to question the policy of venesection under such circumstances again—especially after the suppurative process was established, and when the heart and vascular system were in such a weak and irregular state, as was indicated in the case above. We confess that we should be more inclined to administer stimulants, tonics, and cordials with anodynes, to enable the constitution to bear up against the irritation of the local affection, and the sedative effects of matter draining into the circulation. We should also endeavour, as Dr. Chapman did, to stop the course of the purulent secretion towards the heart, though we doubt whether ligature of the vein, as proposed by our ingenious author, would be the most eligible means of effecting this desirable purpose.

We shall now proceed to some historical notices of inflammation and suppuration of veins, as the result of phlebotomy.

Dr. Chapman has remarked, that " cases of this sort are extremely rare. Few at least have been recorded. I have met in my researches with only one at all analogous." This was the case quoted from John Hunter, at the beginning of this article. In the memoirs of that able pathologist, M. Breschet, we find several cases of this sort, collected from writings to which Dr. C. can have easy access—many of them in his own language. The first case we shall notice is related by Mr. Hodgson, page 512, and may, we think, be acknowledged by Dr. Chapman to be analogous to that detailed by himself, excepting the obliteration of the vessel. It was communicated to Mr. H. by Mr. Broughton, surgeon to the life-guards.

A robust soldier was bled in the arm for ophthalmia. The day after the bleeding, great swelling and pain commenced in the arm, and gradually extended upwards. Fever rose—the pulse was quick and feeble—the breathing was difficult—there was great prostration of strength, with pain in the head, back, and extremities. The symptoms continued until the 23d day, when a painful swelling was observed above the clavicle—and soon afterwards another, beneath the angle of the lower jaw. The symptoms increased slowly—the patient became delirious—and died in the course of the seventh week after the bleeding.

*Dissection.* The cephalic vein had become obliterated from an inch above the wound till it reached the shoulder. The subclavian, axillary, and brachial veins to the bend of the arm were thickened in their coats, so as to resemble arteries. "The external jugular and subclavian veins were filled with pus." When slit open, they were found lined with lymph. The diseased appearances were not gradually lost, but terminated abruptly. The heart was sound.

We think this case is very analogous to that of Dr. Chapman; for as to trifling local differences of appearance in the veins, we do not consider them as altering the general pathology of the disease. Mr. Hodgson remarks:—" several cases of extensive inflammation of veins, after the operation of venesection, have been communicated to me, and I have seen one in which the symptoms and appearances on dissection, although not so extensive, were similar to those which are described in the preceding case. The symptoms were very like those of typhus fever, and the appearances on dissection were, in some places, adhesion and obliteration of the vessels; in others, effusions of coagulated lymph or *pus* into their cavities, with great induration, thickening, and adhesion of the surrounding parts." *On the Arteries*, p. 515.

The following case, related by Mr. Travers, is in still closer analogy with Dr. Chapman's than the one detailed by Mr. Broughton.

Hugh Johnson, aged 33, of impaired constitution, was bled in the median basilic vein of the right arm, on the 15th May, 1810. On the 24th, he complained of pain in the arm, and observed that the orifice had never closed. The pain extended up the arm, and he had a quick pulse. On examination, there was a small open wound, with a slight discharge of pus. Poultices, fomentations, leeches, and purgatives. The local symptoms appeared to diminish, but typhoid constitutional symptoms increased, and he died on the 6th June, or about three weeks from the accident.

*Dissection.* The wounded vein appeared much enlarged, owing to an unusual thickening and adhesion of the surrounding cellular substance. " Internally, pus was found occupying the mediana longa for about two inches below the origin of the median cephalic and basilic, and a similar appearance was traced through the whole course of the humeral vein to the axilla." Before passing under the clavicle, about an inch, the vein abruptly presented its natural appearance, " and there was no sign of disease between that point and the heart." The heart was sound, with the exception of " a small circumscribed patch of lymph upon its anterior surface."

In a case, says M. Breschet, observed by M. Recamier, remarkable for the prompt development of the inflammation, and the speedy death of the patient (on the 8th day) " the cephalic vein, when opened, was found filled with pus throughout its whole extent. The parietes of the vessel were inflamed and thickened—red externally, and of a slightly violet hue in the interior." *Dict. des Sciences Med. vol.* xii. *p.* 344.

We shall conclude this short notice of phlebitis with a case related by M. Breschet, which terminated favourably, though pus was unequivocally demonstrated in the vein.

Jacques Fréaud, 34 years of age, was admitted into the Hotel Dieu, in the month of October, 1814, for an affection of the right arm, resulting from bleeding. It was the 8th day from the accident. The arm was swelled and painful—the orifice where he had been bled was open, and presented a fungous appearance, with discharge of pus. The swelling and pain of the arm were relieved by poultices, fomentations, and rigorous abstinence; and now the vein could be felt, like a cord stretched from the bend of the arm to the arm-pit. M. Dupuytren, to satisfy his pupils that there was inflammation with purulent secretion in the vein, made an opening, about one third way up from the orifice, into the vessel, when immediately there issued forth both blood and matter. Other experiments were made by Dupuytren, which left no doubt on the subject. In about sixteen days the man was discharged from the hospital, cured.

For a great mass of information on this subject, we refer our readers to the 41st volume of the *Dict. des Sciences Medicales, art.* "*Phlebite*"—to the first and third volumes of "*Transactions of a Society* for the Improvement of Medical and Chirurgical Knowledge," where the papers of John Hunter, Matthew Baillie, and James Wilson are to be found—to Mr. Hodgson's work on the Arteries—and to Mr. Travers's Surgical Essays.

---

3. *Diagnosis between Irritation and Inflammation of the Mucous Membranes of the Bronchiæ.** M. Nauche thinks he has discovered a chemical test of bronchial inflammation in the secretion from the mucous surfaces. In the natural state, or where there is merely *irritation*, the mucous secretion he has found to be acid—while, on the other hand, when actual inflammation obtains, the nature of the secretion is changed, and it becomes alkaline. The two states are readily recognized by turnsol paper, which turns red with the one and blue with the other.

" M. Nauche has examined the expectoration, with respect to its acid and alkaline character, in the diseases of the respiratory organs. He believes, from this inquiry, that they may be divided into matter produced by irritation—an increased secretion of the mucous follicles, which line the membrane of the air-passages—and into matter, or expectoration, which results from their inflammation. He has observed that the white, mucous, frothy expectoration, which is frequently brought up in large quantities by persons in a state of agony, has always an acid character, when the air-passages have not been the seat of previous disease. This character is likewise found in the white, frothy expectoration which occurs during the whole continuance of pleurisy, whether acute or chronic; and at the commencement of pneumonia, when the matter expectorated is white, or even yellow. It is often lost in the course of this disease, and re-appears towards its decline. The acid expectoration is likewise found in emphysema of the

* Medical and Physical Journal, Nov. 1824.

lungs and scrofulous phthisis, when the tubercles are but little developed—in the state called crude. It is evident, in all these cases, that the mucous membranes which furnish the expectoration are only in a state of increased excitement, and that they are in no degree inflamed. M. Nauche has likewise found acid expectoration in certain advanced cases of phthisis. He believes that this depended upon these matters being the product of an increased secretion of the mucous membrane lining the excavations formed by the tubercles.

"The expectorated matters, on the contrary, are always alkaline in inflammation of the mucous membrane of the bronchiæ, and in all the cases designated by the names of acute and chronic colds, or mucous phthisis *(phthises muqueuses)*. Although this expectoration is not regarded as purulent, it is nevertheless a kind of pus peculiar to inflammation of these membranes, and analogous to the purulent serosity which is furnished by serous membranes when in a state of inflammation.

"The expectoration likewise becomes alkaline in peripneumony, when the inflammation of the pulmonary tissue communicates itself to that of the mucous membrane of the bronchiæ; and this secretion is the product of the inflammation of these two tissues.

"The expectoration is likewise alkaline in phthisis pulmonalis, in the second or third stage, when the tubercles become broken down. It is usual, in such cases, to find the internal membrane of the lungs deeply altered.

"It frequently happens, particularly among phthisical patients, that both these kinds of expectoration are to be found in the same vessel. That which results from an augmented excitement comes up most easily—is white, frothy, and acid; the other is brought up with difficulty, is yellow, thick, and alkaline. In this disease, when the patient has only the former kind of expectoration, his life is often in the greatest danger." 433.

---

4. *Peculiar Spasms in Children.** The disease to which Mr. North's attention has been drawn, nearly resembles that described by the late Dr. John Clarke, in the fourth chapter of his Commentaries. But it was long ago alluded to by Underwood, and, ten years ago, minutely described by Dr. Kellie, in the 12th volume of the Ed. Med. Journal. In the year 1817, Dr. Johnson published a short paper on the disease (which he denominated "carpopedal spasm") in the 3d volume of the monthly Med. Chir. Journal. Some of these sources of information Mr. North has overlooked. The affection is thus described by the acute observer last mentioned.

"The premonitory symptoms occur at an uncertain age—generally, I believe, between the third and seventh month. At first, they may not be sufficiently striking to attract the particular attention of the friends, although the practitioner, who has had opportunities of watching the

---

* Mr John North. London Med. Journal, January, 1825.

progress of similar cases, might, with much confidence, predict the series of symptoms which is yet to be developed. Each time the child wakes from its sleep, the breathing is for some moments unusually accelerated, and is accompanied by such a kind of noise as would be caused by an increased secretion of the mucus of the aerial passages. If the little patient has previously enjoyed a good state of health, the characteristic rotundity of features observable in the infantile state, will quickly undergo a remarkable change. The countenance soon becomes anxious; the sides of the nose are drawn in; the face is pallid and emaciated. When put to the breast, the child sucks greedily for a moment, but suddenly ceases to do so, and frequently throws back the head, which remains rigidly extended for some time. Whatever may have been the previous condition of the bowels, they now become constipated.

"This state may continue for a very uncertain time, without any remarkable alteration: the following symptoms, however, are gradually added to those above enumerated; they occur irregularly. A convulsive affection of the hand is usually the next morbid sign which excites attention. The child's thumb will be found constantly and firmly pressed upon the palm of the hand. The wrist and ancle joints are bent rigidly inwards. The head is now almost constantly thrown backwards, keeping the anterior muscles of the neck upon the stretch. The inconvenience the child suffers, when he wakes, is no longer confined to a mere acceleration of the breathing. This symptom still continues in an aggravated degree, but the noise accompanying the respiration has gradually assumed a very different character from that which marked it at first: each respiration is now attended by a loud crouping noise, which might be heard in an adjoining apartment. The child has frequent attacks of convulsions, during which the features are much distorted. These convulsive paroxysms vary in violence and in duration in different cases; sometimes the whole body is affected. In the child of a Mons. Lambert, in whom the convulsions were frequent and severe, the state of opisthotonos was so complete, that for many days the head and heels were the only parts which touched the bed: if, with difficulty, this apparently painful position was altered by the mother, it was quickly resumed. The brow of the child is generally knit. The anxiety of the countenance is extreme. There is no febrile action in the system to be detected. No determination of blood to the head is manifested, either by an increase of heat or a flushed countenance.

"I have known the firm contraction of the thumb, the rigidly bent position of the hand and foot, and the crouping noise in respiration, continue for many weeks without intermission. The child sometimes appears lively: its countenance will be animated by a momentary cheerfulness; but it almost invariably awakens from its slumbers, however tranquil they may be apparently, with a convulsive paroxysm, similar to that which I have described. The paroxysm having terminated, the child appears much exhausted, and almost motionless for some time. Dr. Clarke observes, that the term of chronic croup has been sometimes applied to this affection; ‘ but it is very different from croup, and is altogether of a convulsive character.’ ” 40.

Dr. Kellie's description of this curious disease is worth stating in this place, because the pathology of the complaint has given rise to considerable discrepancy of opinion, and the disease itself does not appear to be known to the generality of practitioners.

“ But in many cases this remarkable tumour of the hands and feet has appeared to me to constitute but a part of a disease, of a somewhat more serious and striking nature. Even the swelling itself deserves a more particular description than has been bestowed upon it by Dr. Underwood.

“ It has a considerable degree of roundness and elevation, and looks like that sort of tumour which might arise on the same parts from a blow or contusion. It seems to arise suddenly, as it has generally this roundness and elevation from the time of its first attracting observation. When first observed, it has somewhat of a mottled, livid, and purplish colour; I would say it looks cold like, or that it resembles the chilled hand of a full and healthy child after exposure to a cold and frosty atmosphere. It feels cold also, at least it has no inflammatory heat, and does not appear to be morbidly sensible, or to give any pain to the child when handled. It does not pit on pressure, but rather gives the sensation of firmness and resistance; and when pinched, and attempted to be moved sideways, it has always conveyed to me the notion of a disease of infants, which is known by the name of Skin-bound.

“ This swelling, which is confined to the anconal aspect of the metacarpus of the hand, and to the rotular aspect of the metatarsus of the foot, terminates abruptly at the carpus and tarsus, insomuch, indeed, that, in lusty children, it seems in these places as if confined by a cord or bandage. I have known this tumour to continue for weeks together. Sometimes it disappears in a few days, not again to return. In other instances, it has from time to time disappeared, and reappeared at short intervals. When it continues without abatement for any length of time, for a week or two, it sometimes softens, becomes looser and more compressible; its colour changes to paler, and acquires more of a leucophlegmatic hue; in a few cases it has become truly leucophlegmatic, and the œdema has spread upwards on the legs and thighs. But this is a very rare occurrence, of which I have seen but one or two examples. Its more sudden disappearance, without undergoing these changes, or without passing to the state of leucophlegmasia, is by far more common. But this swelling of the tops of the hands and feet is, according to my observation, connected, in a great proportion of cases, with another appearance of a more formidable nature, a spastic contraction of the flexor muscles of the thumbs in the upper, and of the toes in the lower extremities. The thumb is rigidly contracted, and permanently bent downwards, and laid flat upon the palm of the hand; and, in like manner, the toes are bent down to the plantar aspect of the foot. Along with the thumb, the carpus also is, in some cases, drawn thenard by a spastic contraction of its flexors, which much increases in appearance the prominence and sphericity of the metacarpal tumour. The child labouring under this state of disease is unhappy and restless, but does not appear to suffer much, if any, pain, in the parts affected, unless an attempt be made to force them from their situation; and even this forcing of the thumb or toes from their unnatural position, does not always make the child cry. Though peevish, fretful, and evidently suffering from irritation, he cannot be said to labour under pyrexia at all times. In some cases, indeed, the pulse is more frequent than in health, and towards evening there is an increase of heat, and perhaps flushing of one or both cheeks, with coldness of the feet. The bowels are commonly torpid, and the stools obtained by laxatives are, as Dr. Underwood has described them, fetid and clayey, or green, sour, and slimy. This spasmodic affection, though on some days it may appear more relaxed than on others, is, upon the whole, wonderfully persistent. I have known it continue, with little abatement, for many weeks. In others, it has yielded within a shorter period, in a few days, in a week or a fortnight. In one child, who at length died of supervening marasmus, the contracted state of the thumbs continued, with little occasional relaxations, for three months. The greater number of cases which I have seen have terminated favourably. I have sometimes known eclampsia to supervene to this tonic spasm of the thumbs and toes. One child had repeated fits of convulsion, on three successive days, and eventually recovered. Two others have died after supervening eclampsia. In another case of this disease, in which the thumbs and toes had been contracted for three or four weeks, fever supervened, and the child died after passing through all the symptoms of hydrencephalus acutus. Two other children, affected with the disease in question, have tardily sunk under that train of symptoms which Dr. Armstrong distinguished as the hectic of teething. These are the only instances I have met with, in which this disease has had a fatal termination; and that termination, it may be observed, has been by a sort of conversion, or gradation, by the supervention of eclampsia, hydrencephalus, or abdominal fever." 450.

In all the cases, the age was between six months and two years—consequently within the period of dentition. Dr. K. therefore looks upon dentition as the chief, though probably not the only cause of the disease in question.

Dr. Johnson, in the volume before cited, has related a case, which appears to have been an exquisitely marked one of this peculiar affection—and, as it is short, we shall cite it.

"The patient was a delicate female child, nineteen months old, and cutting three or four teeth at the time. She had been ill eight days before the Reporter saw her; but according to the mother's account, there was little or no variation in the symptoms all the time. When first seen, the child three or four times in the hour was seized with spasmodic affections of the respiratory muscles, consisting of repeated attempts to fill the chest, during which she threw herself back, as in opisthotonos, and appeared as though she would be suffocated. These fits would last ten or twelve minutes, after which the child was somewhat easier, but always fretful and peevish. The backs of the hands and insteps were swoln and hard. The thumbs were rigidly contracted, and locked across the palms of the hands. The toes were bent down towards the soles of the feet, and both wrists and ankles were rigidly bent by the flexor muscles, and kept permanently so. The little patient could take no food: she was slightly feverish; the bowels torpid; and the stools clayey, slimy, and offensive. The eyes looked very heavy and inanimate. The child extremely irritable and restless both by day and by night.

"For reasons unnecessary to mention, no very active means were employed for some days, during which the child got worse, and was evidently declining fast. Hydrencephalus or eclampsia being now dreaded, leeches were applied to the temples; calomel and antimonial powder were exhibited thrice a day, and the warm bath was employed in the evening. After the bath and bleeding, the spasms went off for twelve hours entirely, and the child took some food. They returned, however, but not so violently as before, and the convulsive respiration was much relieved. The calomel and antimony were continued, as was the warm bath; and the gums were deeply scarified wherever a tooth appeared to be coming. From this time the child gradually recovered, and on the 18th day the spasms had entirely disappeared."*

In no instance has Mr. North seen this disease fatal—and we can say the same in our own practice. It is well known, however, that the late Dr. Clarke, and the present most able practitioner, his brother, together with their elèves, looked, and still look upon this disease, or this train of symptoms, as indicative of hydrocephalus, or rather of that state of brain which leads to hydrocephalic effusion. This treatment is consequently the same as in the last-mentioned disease. While we do not coincide with this school in viewing the disease in so grave a light, we think it prudent, in all these cases, to guard the head, while we freely lance the gums, and act upon the bowels. It must be evident that the spasms above described can only be produced through the instrumentality of the brain and nervous system, by which all power is supplied to the muscles, whether within or without the salutary boundary. But a sympathetic affection of the brain and nervous system, as from dentition, bad secretions in the bowels, &c. is a very different thing from an idiopathic affection of the same organ, constituting an inflammation. The *latter* is dangerous, and cannot continue long without fatal consequences—the former (in which we include the subject of this article) is far less dangerous, and may go on, as the cases prove, for some weeks, or even months, and terminate favourably, without any very active measures. Still we would not advise that it should be treated too slightly, but always with the danger of cerebral inflammation or effusion, in our

* Med.-Chir. Journal, for May, 1817, p. 448-9

mind—not in the active manner of acute hydrocephalus, but as a preventive of any such supervention. This appears to be the sentiment of Mr. North and of Dr. Kellie.

"The treatment (says Mr. N.) demanded in the above state, may be very briefly laid down. The gums should be freely lanced, if they appear swollen or inflamed. To keep the bowels open, purgatives must be freely given : calomel, in combination with powder of jalap, is perhaps the best remedy. During the convulsive paroxysm, the child should be put into a warm bath. The diet, if the child unfortunately is deprived of its mother's breast, should be very strictly attended to. The digestive powers are evidently weakened, and it is destructive to impose upon the stomach a task which it is not capable of performing, by giving solid food in any form." 41.

We have now put our readers in possession of all we know respecting the symptoms and treatment of this disease. Its pathology is obscure. For our own parts, we think it is produced by the irritation of dentition, or by morbid secretion in the digestive organs.

---

5. *Dissection of Dr. Henning.* Dr. Henning was one of the principal writers in Hufeland's celebrated Journal, and a man of great learning and considerable talent. He was well known to many medical men of this metropolis, which he visited a few years ago. He died on the 2nd December, 1823, of a chronic and distressing complaint, of which we are unable to give more than a very succinct sketch.

The first symptom was an extraordinary appetite, which being too frequently gratified, was followed by nausea, for the removal of which the patient was in the habit of having recourse to emetics of tartarized antimony. Obstinate and almost indomitable constipation was the next phenomenon, and was little relieved by any medicine employed. Being seized with typhus fever, he went through that disease with safety, and the condition of the digestive organs was, for a time, improved. In 1817, after some depressing moral causes, and exposure to atmospherical vicissitudes, he became jaundiced, and affected with a diarrhœa, which gave way to the usual remedies, and his health was apparently restored, at least, for a short time. Constipation again succeeded, and was of the most obstinate nature. Then came deep-seated pain of the epigastrium, which only gave way when vomiting was excited. Still the appetite continued enormous, and the patient persisted in satisfying it, and that often with the most indigestible substances, though emetics were indispensible consequences. Worn out by canine appetite, pain, sickness, and constipation, this unfortunate physician was released from a painful state of existence, at the period abovementioned.

*Dissection.* The stomach was of enormous size, and in the neighbourhood of the pylorus, its coats were thickened, and of the consistence of cartilage. Every where else, the coats of this organ were exceedingly attenuated. There were no other morbid appearances of any importance in the abdomen or other cavities of the body. *Journ. der Pract. Heilkunde, Aug.* 1824.

---

6. *Fatal Asthma from Affection of the Glottis.** The researches of Magendie and others, teach us that, 1st—the constrictor muscles of the glottis are supplied by filaments from the superior laryngeal nerve—whilst the dilator muscles are supplied by branches from the inferior laryngeal or *recurrent* nerve—2nd, that the glottis dilates during inspiration, and is closed, almost hermetically, when we make any considerable muscular exertion, as in lifting a heavy weight, for example. Every one must have observed that the breath is firmly retained when we make any great straining exertion with the muscles, especially of the upper extremities. These physiological points are requested by our author to be borne in mind, as they will come in to an explanation of the following case.

*Case.* Michel, 22 years of age, a servant, was received into the hospital Cochin, the 26th of June, 1822, having been discharged only eight days previously from a MAISON DE SANTE, where he had been under treatment for two months, for what was called a putrid fever. The following symptoms were now observable; viz. considerable hoarseness—violent pain in the throat—great sense of suffocation on the least muscular exertion—tongue white—appetite good—pulse frequent and small—face pale and emaciated—chest sonorous, except at the inferior part of the right side—respiration audible in all parts of the thorax—nothing remarkable in the action of the heart. The same symptoms were observable the following day. He expectorated abundantly. At ten o'clock in the evening he was obliged to start from his bed—his inspirations being profound and frequent, accompanied by a peculiar hissing noise. The patient appeared terrified, his eyes starting, and dreading instant suffocation, which indeed appeared inevitable. His pulse was scarce perceptible. These alarming symptoms abated a little in the night. *June* 28. Still great anxiety. Blister to the region of the larynx. In the course of the day, a return of the sense of suffocation, with a horrible state of anguish, during which the patient tore the blister off his throat, and appeared strangling—his head erect, and his neck stretched as much as possible. 29th. Sense of strangulation still more distressing than ever—he was constantly putting his hand to his throat, as if to remove something that was strangling him. He cried for prompt relief from his dreadful anguish, his eyes starting from their sockets, and his countenance the image of despair—the whole of the respiratory muscles in a state of convulsive orgasm. These horrible symptoms continued till four o'clock in the morning of the 30th, when death put an end to his sufferings!

*Dissection.* An abscess was found situated at the posterior part of the larynx, and also on the sides, the cavity of which was capable of

---

* Dr. M. J. Bouillaud. Journal Complementaire, Juillet 1824.

containing a filbert, and its internal surface smooth. The cricoid cartilage was denuded. On closer examination, it was found that the abscess extended round the cricoid cartilage. The crico-arytenoid muscles were of a greenish colour. The arytenoid cartilages were confounded with the muscles abovementioned, which were in a state of disorganization. No nerves could be traced in them. The arytenoid muscle, however, and its nerves were well preserved. The glottis was not above three or four lines in length (its rima.) The articulations of the arytenoid cartilages with the cricoid were destroyed. The mucous membrane of the larynx was pale, and the cavity itself filled with reddish and frothy mucus, which was also found in the trachea and bronchia. The mucous membrane of the lungs was, generally speaking, of a deep red colour. The inferior and posterior portion of the right lung was hepatized, but all the rest of the lungs sound. There was no disease in any other part of the thorax. *In the small intestines were found numerous ulcers perfectly cicatrized.*

We shall not enter into the detail of M. Bouillaud's attempt at explaining the phenomena of strangulation in this case. It is thus, that the nerves and muscles of dilatation about the glottis were diseased or destroyed, while those of *constriction* were in a state of integrity. The medical attendants were completely puzzled as to the nature of the case, before the death of the patient; but surely there was the clearest evidence that obstruction to respiration existed at the upper part of the trachea, and, therefore, the operation of tracheotomy was so loudly called for, that we are astonished any surgeon could allow his patient to struggle two or three days in a state of strangulation, without performing the operation!—It was in vain that M. Bouillaud and his associates ascertained, by the stethoscope and percussion, that nothing material was wrong about the lungs and heart, when they stood listlessly looking on, and suffered their patient to die the most horrible death that human nature can undergo. The disease of the larynx might, or might not be, afterwards, fatal. That chance should have had no effect in deterring from tracheotomy, which would have given present relief, and a probability of permanent security. Our author cites a case that happened in one of the wards of La Charite, under M. Fouquer, which is completely in point. A young woman had all the symptoms of laryngeal phthisis, and was on the point of suffocation, when M. Roux practised tracheotomy. The operation was attended with some alarming symptoms at the time, but they gradually subsided, and the young woman perfectly recovered. M. Bouillaud observes that, as the dilator muscles, in this case, were in a state of disorganization, it is probable that it would have been necessary to keep an artificial passage open for life. —"One of the most deplorable events that can happen to a man, says he, is, without doubt, the loss of *voice and speech*." We are very far from agreeing with Dr. Bouillaud on this point. We do not think that the said loss is by any means the most deplorable that can happen to man—or woman either. The patient who was operated on ten years ago, and more, at Portsmouth, and who still breathes through a tube in the trachea, is in perfect health, and the full enjoyment of life—with the exception that he can only listen to, but not join in the conversation around him. It would be the part of a wise man to pursue this plan often, without any silver tube in his throat.

There is a horse at Charenton, which had tracheotomy performed on him, and who breathes through a tube. He draws a chaise; and this fact seems to surprise M. Bouillaud, for the physiological reason mentioned at the beginning of this article. But our author ought to recollect that, although we stop the breath when we wish to make any violent muscular effort, it is not absolutely necessary that the rima glottidis should be closed during very great muscular exertion. A horse running with all his might, or a man either, keeps the glottis open all the time.

We hope our surgical brethren will attempt, more frequently than they do, to save the lives, or at least, the sufferings of patients threatened with suffocation in laryngeal phthisis, by tracheotomy.

---

7. *Remarkable Intus-susception.* Mr. Davies has related (*Med. Repos. Dec.* 1824.) a curious case of intus-susception, where one half of the colon was received into the other half, and where the caput coli had gone down right to the anus. "The intus-susception had evidently commenced at the *caput cœci*, and as it extended, it doubled the colon upon itself, until the part which was first received into the portion below it, arrived at the situation already mentioned." The case occurred in a child, six years of age, who, having eaten a quantity of raw carrots, was seized with pains in the bowels that continued more or less for eight months afterwards, when she died. The bowels, in the mean time, were regularly open, about three times a day; "but the stools were always very thin and watery—and during the last three months, they were mixed with slime and blood." As the lower half of the colon was lined with the inverted upper half, it followed that no part of the fœcal remains came in contact with the colon at all—the termination of the ileum being drawn down through the centre of the intus-susception to the anus. This may account for the stools being thin and watery. There was no remora in the large intestines for the formation of natural stools.

---

8. *Hospital Reports.** We noticed the first of these Hospital Reports (from the Wards of Laennec) in our third number, page 240 *et seq.* We now proceed to take a short view of the second of these returns, which is made out by M. Bayle, from the ward of Professor Cayol. Our author commences with some account of those that remained in

---

* Des Maladies Observées à la Charité, dans les Salles de Clinique de M. Le Professeur Cayol—(April to May, 1824) par M. Bayle. *Revue Med. November*, 1824.

Hospital when Laennec's quarter closed. It is stated that among these there were no less than five recoveries from what was considered, and by the stethoscope ascertained to be—*confirmed phthisis.*

There had been cough—purulent expectoration—progressive emaciation—hectic fever—nocturnal perspirations—and, finally, pectoriloquism in some part or other of the chest.

In this report there is the sequel of a remarkable case, which we have given in pages 241-2 of our third number. It was of a young man who had entered the hospital with articular rheumatism, which gave way to antimonials. Next succeeded pleuro-pneumony, with great action of the heart, which led M. Laennec to examine that organ, and he found it organically diseased. The pulmonic inflammation was cured—but hypertrophia of the heart remained and went on increasing. At length, it was found that every large artery in this patient's body presented a hissing noise. Such was his state when our former notice closed. Some time afterwards, the patient was seized with furious delirium which ceased in five or six days, after the usual remedies, as cold to the head and sinapisms to the feet. To the delirium succeeded a great prostration of the intellectual faculties, in which condition the patient continued about a month, with this remarkable peculiarity, that when he became refractory, the action of the heart diminished, and the hissing noise of the arteries subsided, and *vice versâ.* Diarrhœa now came on—the patient wasted to an extreme of emaciation, and appeared in the jaws of death. In this state M. Cayol tried various means of recalling the rheumatic inflammation to the joints, as sinapisms, &c. without effect. Nevertheless, after six months sojourn in La Charité, the patient gradually recovered his reason, and with it his health—the action of the heart returning to its natural rhythm.

The above case is remarkable as having completely deceived Laennec with the stethoscope in his hand. He pronounced the disease of the heart to be organic, and consequently incurable—for where do we see organic lesion of this viscus cured? It is a dangerous thing to give a very decided prognosis or diagnosis in diseases of internal organs, since one failure will do more harm than fifty instances to the contrary. The worst of the stethoscope is, that the confidence which the auscultator places in it, may occasionally take off his attention from the general phenomena of the disease, by which others are guided in their diagnosis. Let him, therefore, be on his guard against this failing, lest he bring a useful diagnostic auxiliary into contempt.

We now come to the patients who had entered the hospital during M. Cayol's own receiving quarter. They were, in number, 182, of which 111 were acute, and 71 chronic affections. There were 37 of continued inflammatory fever, arising from no appreciable cause, and occurring almost entirely among the labouring classes. Excepting where the patients had used stimulating ingesta at the beginning of the disease, there were very few symptoms of gastric or intestinal irritation—the head or chest appearing to be the chief seats of local affection.

On entering the hospital, M. Cayol placed the fever patients on the lowest diet—ordered them diluent drinks, emollient glysters, fomentations, &c. He applied leeches to the part which seemed most affected, as to the pit of the stomach, the temples, or the chest. Care was taken, however, to distinguish those gastric and intestinal affections which were symptomatic merely of cerebral disturbance, from those which were primary. In elucidation of this discrimination, he relates the heads of three cases. *Case* 1. A young man, 20 years of age, had acute cephalalgia; very red tongue; great tenderness at the epigastrium on the slightest pressure; frequent nausea and vomiting. Here then were the Broussaian signs of gastro-enterite or, at all events, gastro-enteritic irritation. Next day, the head-ache was increased, the pupils dilated, with delirium and constant vomiting. Fifteen leeches applied to the temples, and cold applications to the head completely removed not only the cerebral, but the whole of the gastro-intestinal symptoms. The *second case* was that of a young woman who was affected and treated in similar manner to the above, and with similar success. The *third case* analogous.

Of the 37 cases of fever, two only proved fatal:—one might be termed *adynamic*, the other, *ataxique*. The first was an individual debilitated by fatigue, bad diet, and mental anxiety. The fever was preceded by rheumatism, which at first was seated in the hepatic region and edges of the ribs, accompanied by cough, tenderness of the hypochondrium, head-ache, and yellow tinge of skin. *Whey and gum-water were prescribed, and a dozen of leeches were applied to the anus.* These means produced a sensible amelioration at first. On the 4th day, the hypochondriac pain ceased entirely, giving place to a very painful swelling of the left shoulder. On the 5th day, delirium supervened, with nausea, vomiting, and diarrhœa; pulse small and weak. *Sixth day.* Augmentation of the diarrhœa; cessation of pain in the left shoulder; bad smell about the patient; decomposition of the features; sinking of the eyes—decubitus dorsalis, &c. *Some extract of bark with tincture of rhubarb was prescribed, and a sinapism to the left shoulder. Seventh day.* The left shoulder is swelled and painful, as are the right elbow and wrist—cessation of the diarrhœa—great amelioration of the cerebral symptoms—more expression in the countenance. This favourable train of phenomena did not continue long. The articular swellings subsided, and all the bad symptoms returned, except the diarrhœa. Three times, in the course of this fever, did these metastases take place from the external to internal parts; and thrice was the rheumatic inflammation recalled to the surface by tonics internally, and rubefacients externally. On the 12th day, the patient's intellects were perfectly clear, and he received the sacrament; but debility had now made great progress—and the patient sunk on the 16th day, preserving his intellectual faculties in a state of integrity till the last moment.

On dissection, the joints which had been the seat of the rheumatic pain were found inundated with a puriform fluid. On the mucous membrane of the stomach and bowels were a few rosy-coloured spots, (such as here seen frequently in those who have had no disturbance of digestive functions during life) but without any alteration whatever of structure. The thoracic organs were sound, as was the brain, with the exception of a slight injection of the meninges.

The following case is not much in favour of the Broussaian mania now raging over Europe.

"A woman, about 50 years of age, was carried to La Charité, in the 23d day of a very severe fever, and apparently in a dying state. The marks of numerous leech bites, blisters, and sinapisms, attested the activity of the treatment previously pursued. The symptoms were those which characterise the last stages of typhoid fevers. *Acidulated decoction of bark, with musk, were prescribed,* and she rallied a little, so that she lived six days after her entrance into the hospital."

On dissection, ten hours after death, the body was found to be greatly advanced in putrefaction, and very much swelled. "The encephalon and thoracic organs were sound. The stomach and intestine were filled with gas. The mucous membrane lining them was pale and fine; and after the most scrupulous examination, presented not the slightest trace of alteration." The author remarks, and with justice, that "this dissection, made in the presence of numerous students (most of whom expected to see a train of very different phenomena) incontestibly proves that the group of symptoms to which has been attached the name of *adynamic fever*, may exist without any appreciable local inflammation, even of the mucous membrane of the stomach or bowels."

M. Cayol, we are informed, endeavours to impress on the minds of his clinical pupils, the following pathological precepts and doctrines.

"1^mo. It is undeniable," says he, "that the progress of pathology has lessened the number of essential or idiopathic fevers—and that many which have been ranged in this class, were merely symptomatic of local inflammation, especially of the cerebral and abdominal organs. But at the same time, the existence of idiopathic fevers is equally proved, since we have become better acquainted with the symptoms which characterize local affections. Idiopathic fevers have a physiognomy peculiar to themselves, which is not to be confounded with that of symptomatic fevers. The local affections which are observed in their course, are not at all in correspondence with the intensity of the general symptoms. They are sanguineous or serous congestions rather than veritable phlegmasiæ.

"These local affections do not precede the fever, but, in general, take place in the advanced stages of it—hence they cannot be the cause but the effect of the fever. The phenomena of idiopathic fevers are more in correspondence with those of the exanthemata, than with those which are symptomatic of local inflammation,"

*Secondly.* M. Cayol observes that, in the great efforts which have been made, in latter times, to explain all the phenomena of fever on the principle of inflammation of the mucous membrane of the stomach and bowels, *effects* have frequently been taken for *causes.* The redness of the tongue, on which so much has been said, is, like the heat of the skin, one of the primitive phenomena of fever. Whenever, either from external or internal, moral or physical causes, we have quickened circulation and respiration—that is, whenever we have fever, we observe almost immediately *that*, with more or less of dryness in the skin—which febrile heat coincides always with an analogous state, not only of the gastro-intestinal lining membrane, but of all the mucous membranes. Whenever the skin is hot and dry, the tongue is red. And when there is redness of the tongue, with thirst, anorexia, and epigastric sensibility, there is also heat and malaise in the aerial passages and chest—dryness of the pituitary membrane, redness of the urine—lassitude and pain in the region of the kidneys. "All these phenomena evidently depend on the same cause—namely, a turgescent state of the capillary system of the skin and mucous membranes in the reaction of fever. Fever, therefore, is no more a gastro-enteritis, than it is a bronchitis, a cystitis, or a nephritis."

*Thirdly.* While, in consequence of this febrile movement, the skin and mucous membranes become the seat of strong sanguineous congestion and augmented sensibility, the slightest irritating causes may there determine inflammation. Hence the necessity for a mild temperature of the air and rigid abstinence, during the early stage of fever, or period of excitement. If this be unattended to—if irritating substances be taken into the stomach, as is too often the case, they readily kindle up an inflammation there—an inflammation which may become sufficiently intense to produce a symptomatic fever that alters entirely the original disease. Such, according to M. Cayol, is the origin of most of the gastro-enterites which are met with in the hospitals. The following may account for the greater frequency of these inflammatory affections of the mucous membranes in France than in England. "On sait que les gens du peuple ne manquent presque jamais, dés les premiers jours de la fièvre, de s'administrer *du vin chaud ou d'autres boissons excitantes.* Ils reussissent quelquefois à provoquer une sueur abondante qui fait avorter la maladie ; mais le plus souvent ces boissons excitantes determinent un certain degré d'inflammation de la membrane muqueuse des voies digestives."

When a fever patient arrives at the hospital with symptoms of gastro-intestinal irritation, M. Cayol interrogates him respecting the regimen which had been used since the beginning of the disease ; and in general it is found that the fever has preceded the local inflammation, and that this last is the result of direct excitation. It is in these last cases *only*, (which, however, are sufficiently numerous) that the professor has recourse to leeches to the anus and epigastrium.

Where, on the other hand, no improper regimen has been pursued in the beginning of the disease, the redness of the tongue is observed to be in correspondence with the heat of the skin, and decreases, pari passu, with the latter, without the assistance of leeches. When the symptoms of general plethora are such as to demand sanguineous evacuations, M. Cayol prefers venesection to leeching.

But if improper regimen, at the commencement of fever, frequently determines irritation or inflammation of the internal surface of the bowels, so, on the other hand, the inhalation of cold air or irritating vapours, not unfrequently produces irritation or inflammation of the mucous membrane of the air passages—a far more formidable complication than that of gastro-intestinal phlogosis. When catarrhal inflammation supervenes in the course of idiopathic fever, there is this singularity attending it—namely, that it does not ordinarily shew itself in the form of cough, but only in the shape of acceleration and tightness of the breathing, and by the mucous rattling (râle muqueux) ascertained by auscultation. "Without this last mean of exploration, we could not, in the majority of cases, recognize this dangerous complication in fever."

Of the treatment of continued fever on the continent, we need not speak, in this place, as it is well known to our readers; but the foregoing extracts will shew that the doctrines of Broussais are viewed in a proper light by his compatriot Cayol. This indeed is the light in which they will be held when their author is no more, and when time shall settle the exact estimate of their value.

*Intermittent Fevers.* Nine of these were admitted, and gave way, as usual, to the sulphate of quinine, in the quantity of from 12 to 18 grains in the apyrexial interval.

Of the chronic diseases we do not see any in this report that need arrest our attention.

---

9. *Tympanitic State of the Pericardium.* Mr. Scott of the Haymarket, aged about 47, had, for three or four years, been declining in health, but had not been under regular medical superintendence till a few weeks before his decease. No regular history of the complaint, therefore, could be obtained. He stated, however, that his appetite and strength had gradually declined, but his chief complaint was a fluttering, a palpitation, and a sense of anxiety about the region of the heart, with disturbed sleep, and frightful dreams. When seen a few weeks before death, his countenance was like that of a person in a state of anemia, except that there was also a chlorotic tinge in the skin. The pulse was very feeble, quick, and irregular—there was a tendency to œdema about the ancles—the appetite was almost entirely gone, and the patient felt approaches to syncope, on using any exertion, or ascending stairs. His mind was desponding, and temper irritable. The motions from his bowels were perfectly healthy. The chest in every part sounded remarkably well. In the region of the heart, percussion elicited as clear a sound as in any other part of the chest. The impulse of the heart against the ribs was very feeble, and scarcely audible. It was also irregular, in correspondence with the state of the pulse. The patient was under the care of Mr. Fincham of Spring Gardens, and was visited successively by Dr. Hooper, Dr. Macmichael, and Dr. Johnson.

When the above symptoms and phenomena were ascertained by the last-mentioned physician (not in consultation with the others) he gave it as his opinion to Mr. Fincham, that the patient laboured under disease of the heart—and that the nature of the lesion was probably a weakening and degeneration of the muscular structure. In a few days after this examination, the patient suddenly died, and the body was examined by Dr. Johnson, Mr. Fincham, and Mr. Henry Johnson.

*Dissection, 13th February*, 1825. The body extenuated, but still there was some peculiarly yellow fat on the chest and abdomen. The muscles, though wasted, were of a vivid red colour. All the organs in the abdomen were sound. On opening the chest, the lungs presented a beautiful blue appearance, sparingly mottled with white; they were very sound. Between the two lungs, there presented itself a pellucid membrane distended with air. This was found to be the pericardium, reduced to a most extraordinary degree of tenuity, and distended with a very considerable quantity of air. The heart was small (not half filling the pericardium) and extremely degenerated in substance, a great part of its muscular structure being converted into a kind of fat. The whole was so lacerable as scarcely to bear handling. The parietes of the left ventricle were not more than a quarter of an inch in thickness—the internal surfaces of the cavities were pale, wasted, and not containing a single drop of blood. Neither was any blood to be seen in the large vessels issuing from the heart. There was nothing particular in the valvular structure of the organ.

Dr. Johnson has met with several cases where the heart was in this degenerated condition, but never before observed such a distention of the pericardium by air. It is quite evident, from the size of the pericardium, and its extreme tenuity, that this collection of air must have been of some standing. This phenomenon accounts for the region of the heart being as sonorous as any other part of the chest, which is not usually the case.

